# Melanogenesis Inhibitory Activity of Two Generic Drugs: Cinnarizine and Trazodone in Mouse B16 Melanoma Cells

**DOI:** 10.3390/ijms12128787

**Published:** 2011-12-02

**Authors:** Te-Sheng Chang, Victor Chia-Hsiang Lin

**Affiliations:** 1 Department of Biological Science and Technology, National University of Tainan, 33 Sec. 2 Su-Lin St., Tainan 71702, Taiwan; 2 Department of Urology, E-Da Hospital, Kaohsiung 84001, Taiwan; E-Mail: victorlin0098@gmail.com; 3 The PhD Program of Biotechnology, Institute of Biotechnology and Chemical Engineering, I-Shou University, Kaohsiung 84001, Taiwan

**Keywords:** cinnarizine, inhibition, melanogenesis, trazodone, tyrosinase

## Abstract

More than 200 generic drugs were screened to identify the inhibitory activity on melanogenesis in mouse B16 melanoma cells. Cinnarizine and trazodone were identified as melanogenesis inhibitors. The inhibitory effects of the two drugs on cell survival, melanogenesis, and tyrosinase activity were investigated. The results showed that both cinnarizine and trazodone inhibited melanogenesis in B16 cells by a dose-dependent manner at the non-cytotoxic concentrations. Based on the results of the present study, seeking new melanogenesis inhibitors from generic drugs is an alternative approach to developing new depigmenting agents in cosmeceuticals. Moreover, cinnarizine and trazodone were proven to be good candidates as skin-whitening agents for treatment of skin hyperpigmentation.

## 1. Introduction

Human skin color is mainly determined by pigment melanin, which is produced by dermal melanocytes through a melanin synthesis process, melanogenesis. Melanogenesis occurs in a special organelle, melanosome, in melanocytes and is initiated by a key enzyme, tyrosinase [1]. The enzyme catalyses the first step of oxidation of l-tyrosine or l-DOPA (l-3,4-dihydroxyphenylalanine) to dopaquinone, which would form melanin in advance. This is a rate-limiting step in melanin synthesis because the remainder of the reaction sequence can proceed spontaneously at a physiological pH value. Although melanin mainly plays a photoprotective role, the accumulation of abnormal amounts of melanin in different parts of the skin, which results in pigmented patches of skin, might become an esthetic problem. Therefore, several studies have focused on the inhibition of tyrosinase activity and the prevention of abnormal pigmentation [2–4].

Recently, a huge number of melanogenesis inhibitors were discovered due to a great improvement on knowledge about the regulation of melanogenesis [5–7]. However, few of them have been given approval in cosmeceutical ingredients due to a lack of clinical trials to demonstrate the safety of the newly discovered drugs on human usage. In fact, both time and cost for the clinical trials on safety are usually longer and higher than those for the trials on skin-whitening efficiency. Based on the limitation of safe usage for new-drug development, we try to find new skin depigmenting agents from generic drugs which have been used in hospitals for therapy on other diseases. These drugs have been demonstrated to be safe and approved for human usage. As long as the generic drugs are proven to possess depigmenting activity, they could be used in cosmeceuticals very soon without the necessity for clinical trials on safety.

**Figure 1:**
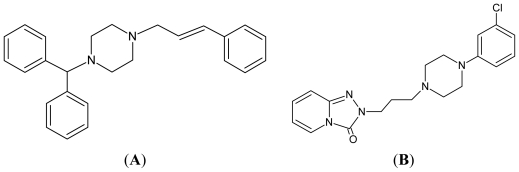
The chemical structures of cinnarizine (**A**) and trazodone (**B**).

In the preliminary study, we selected more than 200 generic drugs to search new depigmenting agents. The criteria of the selected drugs include being approved by The Food and Drug Administration of Taiwan, on the paid-drugs list by The Bureau of National Health Insurance of Taiwan, and in the form of pill. The list of the drugs is shown in Table 1. Six drugs, including aspirin, cinnarizine (Figure 1(A)), danazol, homochlorcyclizine, miconazole, and trazodone (Figure 1(B)), were identified as melanogenesis inhibitors. Among them, aspirin [8] and miconazole [9] have been reported with the anti-melanogenesis activity in B16 cells in the literatures. We also have studied the melanogenesis inhibition by danazol [10] and homochlorcyclizine [11]. In the present study, we continued to investigate the melanogenesis inhibition by cinnarizine and trazodone in B16 cells.

## 2. Results and Discussion

**Figure 2:**
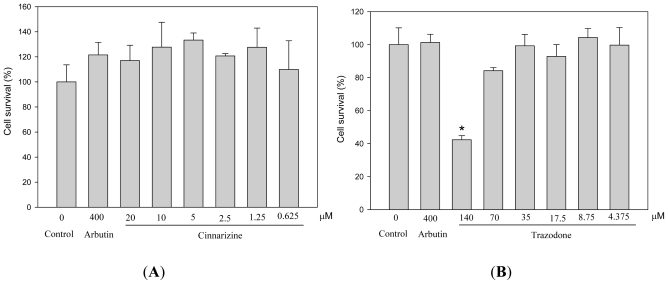
Effects of cinnarizine (**A**) and trazodone (**B**) on cell survival in B16 cells. The cells were cultivated for 1 d and then stimulated with 100 μM of IBMX for 2 d with various concentrations of the tested drugs. The cell survival was determined by the MTT method. Averaged data (n = 3) are presented with error bars indicating SD. A value of *p* < 0.001 (*), obtained with a Student’s *t*-test by comparing the data with that of control, was considered statistically significant.

We evaluated the cytotoxicity of cinnarizine and trazodone to mouse B16 melanoma cells before the depigmenting assay. The cells were treated with different concentrations of the drugs and then the cell survival was determined by the MTT method (Figure 2). Trazodone showed significant cytotoxicity at 140 μM, while cinnarizine did not exhibit any cytotoxicity less than 20 μM. In order to avoid the misinterpretation of depigmenting results from killing melanocytes but not from inhibiting melanogenesis by the drug, we used 70 μM and 20 μM as a maximal concentration for trazodone and cinnarizine, respectively, in the following experiments.

**Figure 3:**
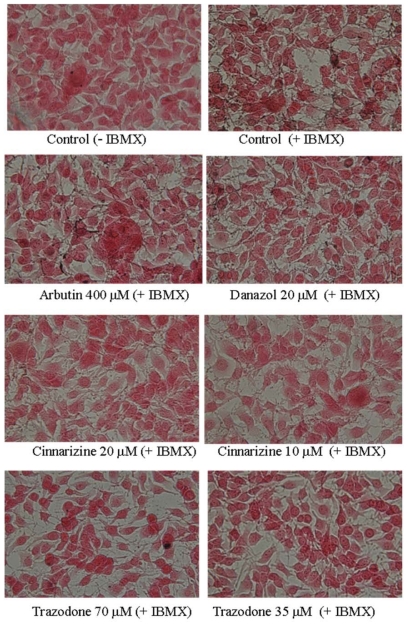
Fontana-masson stain of B16 cells treated with cinnarizine and trazodone. The cells were cultivated for 1 d and then stimulated with 100 μM of IBMX for 2 d with various concentrations of the tested drugs. The melanin content of the cells was determined by Fontana-Masson stain, as described in the experimental section.

**Figure 4:**
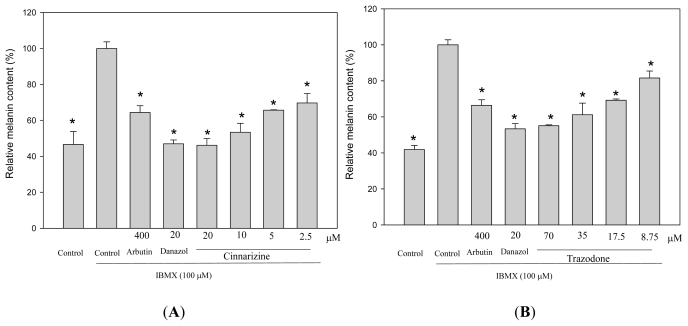
Effects of cinnarizine (**A**) and trazodone (**B**) on melanogenesis in B16 cells. The cells were cultivated for 1 d and then stimulated with 100 μM of IBMX for 2 d with various concentrations of the tested drugs. The melanin content of the cells was determined by spectrometry, as described in the experimental section. Averaged data (n = 3) are presented with error bars indicating SD. A value of *p* < 0.001 (*), obtained with a Student’s *t*-test by comparing the data with those for the IBMX-stimulated control, was considered statistically significant.

To study the effects of cinnarizine and trazodone on melanogenesis in B16 cells, the cells were treated with the drugs and the melanin contents in the treated cells were directly monitored by Fontana-Masson stain of the cells (Figure 3). In the present study, we used IBMX, which is an elevator of cellular cAMP level, to stimulate melanogenesis in B16 cells. Two melanogenesis inhibitors, arbutin and danazol, were used as positive standards [10]. The melanin content in the cells was increased after IBMX treatment and the increased in melanin content was reduced by both arbutin and danazol treatments. All cinnarizine and trazodone treatments also significantly decreased the melanin content of the treated cells compared with that of the IBMX-stimulated cells. The melanin content of the treated cells was also determined by a photometric method, which detects the melanin content in cells via the absorption of the NaOH-dissolved melanin at 490 nm (Figure 4). The resulting profile was similar to that obtained with Fontana-Masson stain and dose-dependent melanogenesis inhibitions by the two drugs were clearly observed.

**Figure 5:**
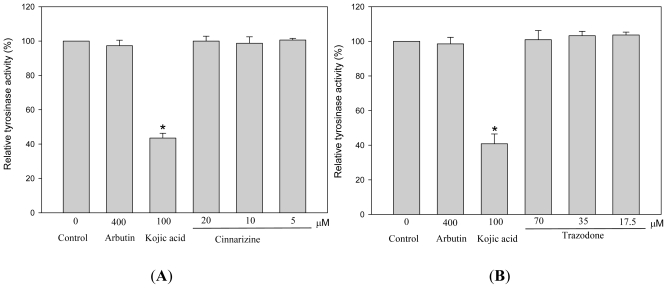
Effects of cinnarizine (**A**) and trazodone (**B**) on murine tyrosinase activity. Tyrosinase activity was determined by spectrometry, using l-DOPA as a substrate, as described in the experimental section. B16 cells were cultivated for 5 d and lysed to obtain a crude tyrosinase extract. The cell-free tyrosinase activity was determined by directly mixing the crude tyrosinase, l-DOPA, and various concentrations of cinnarizine and trazodone. Averaged data (n = 3) are presented with error bars indicating SD. A value of *p* < 0.001 (*), obtained with a Student’s *t*-test by comparing the data with that of control, was considered statistically significant.

Because tyrosinase plays a key role in melanogenesis, we then investigated the effects of the two drugs on the activity of this enzyme using a photometric method (Figure 5). Unexpectedly, neither cinnarizine nor trazodone directly inhibited tyrosinase activity, while kojic acid, a standard of tyrosinase inhibitor, showed strong inhibition on the enzyme activity. Therefore, melanogenesis inhibition by cinnarizine and trazodone in B16 cells apparently did not occur via direct inhibition on tyrosinase activity and the two drugs are not tyrosinase inhibitors. The detail molecular mechanism of the melanogenesis inhibition by cinnarizine and trazodone needs to be resolved in a further study.

A previous study has reported that many cytokines and growth factors play important regulatory roles in melanogenesis [12]. α-Melanocyte stimulating hormone (α-MSH) is the most well-studied hormone. This hormone binds to its receptor, melanocortin receptor 1 (MC1R), on the membrane of melanocytes and stimulates melanogenesis via the GPCR (G protein-coupled receptor)-cAMP-MITF (microphthalmia-associated transcription factor) pathway where the melanogenesis-related enzymes, including tyrosinase and tyrosinase-related proteins 1 and 2 (TRP1 and TRP2) are upregulated. Accordingly, agents blocking the signal pathway would also exhibit depigmentation against melanocytes [5–11]. It is very interesting to note that the functions of four discovered melanogenesis inhibitors are related to hormone regulation. Both cinnarizine and homochlorcyclizine are histamine receptor antagonists. Trazodone is a serotonin receptor antagonist and danazol is a synthetic testosterone. These four generic drugs were proven to inhibit IBMX or α-MSH-stimulated melanogenesis in B16 cells. In our previous studies, we have demonstrated that homochlorcyclizine and danazol inhibited α-MSH-stimulated melanogenesis in B16 cells, but not via antagonism toward the histamine receptor or androgen receptor, respectively [10,11]. Until now, how these generic drugs affect the α-MSH signal pathway and then inhibit melanogenesis remains unclear and needs to be studied in the future. In addition, despite these newly discovered drugs that showed potent antimelanogenic activity, more clinical assays should be performed before development as depigmenting agents and the related *in vivo* assays that were under out current work.

## 3. Experimental Section

### 3.1. Materials

All generic drug tablets used in this study are listed in Table 1 and were obtained as gifts from Pin-Chin Tsai in Department of Pharmacy, E-Da Hospital, Kaohsiung, Taiwan. Arbutin, 3-(4,5-dimethylthiazol-2-yl)-2,5-diphenyltetrazolium bromide (MTT), l-DOPA, dimethyl sulfoxide (DMSO), trypsin/EDTA, synthetic melanin, 3-isobutyl-1-methylxanthin (IBMX), cinnarizine and trazodone were purchased from Sigma (St. Louis, MO, USA). All other chemicals were obtained from Tokyo Chemical Industry (Tokyo, Japan).

### 3.2. Cell Cultures and Drug Treatments

Mouse B16 melanoma cells (4A5) were obtained from the Bioresources Collection and Research Center (BCRC, Food Industry Research and Development Institute, Hsinchu, Taiwan). The cells were cultured in Dulbecco’s modified Eagle’s medium (DMEM) supplemented with 10% (v/v) fetal bovine serum at 37 °C in a humidified, CO_2_-controlled (5%) incubator. The cells were seeded at an appropriate cell density in a 24-well plate. After 1 d of incubation, the cells were treated with various concentrations of the drugs in the absence or presence of a stimulation agent (100 μM of IBMX) for another 2 d. Thereafter, the cells were harvested and used for various assays.

### 3.3. Measurements of Cell Viability

MTT assay was performed to examine the viability of cells [7]. Afterwards, the cells were incubated with the samples for 48 h, the culture medium was removed and replaced with 1 mg/mL MTT solution dissolved in phosphate-buffered saline (PBS) and incubated for an additional 2 h. The MTT solution was then removed and DMSO was added, following which the absorbance of the dissolved formazan crystals was determined at 570 nm by a spectrophotometer.

### 3.4. Fontana-Masson Stain

At the end of cell culture, the cells were harvested and washed twice with PBS. Fontana-Masson stain of the cells was conducted by a Fontana-Masson stain Kit (ScyTek Lab., Logan, UT, USA) according to the manufacturer’s instructions. The Kit is used for the visualization of melanin in cells, where cell nuclei, cytoplasm and melanin would display red, light pink, and black, respectively, after staining. The staining cells were photographed under a phase-contrast microscopy equipped with a digital camera.

### 3.5. Determination of Melanin Content

The assay of evaluation of melanin content in B16 cells was according to our previous paper [7]. At the end of cell cultivation, the cells were harvested and washed twice with PBS. The pelleted cells were lysed in repeated frozen in lysis buffer containing 20 mM sodium phosphate (pH 6.8) and 1% Triton X-100. After centrifugation at 15,000 × g for 15 min, the melanin pellets were dissolved in 1 N NaOH containing 20% DMSO for 1 h at 95 °C. The melanin content was measured by the absorbance at 490 nm.

### 3.6. Measurements of Tyrosinase Activity

Murine tyrosinase activity was examined by measuring the rate of oxidation of l-DOPA [7]. A source of crude cellular tyrosinase was obtained by homogenizing B16 cells in 20 mM sodium phosphate (pH 6.8), 1% Triton X-100, and 1 mM PMSF at 4 °C with 30 repeated strokes in a Dounce homogenizer. Detergent was used to release the membrane-bound tyrosinase from the melanosomes. The lysates were centrifuged at 15,000 rpm for 15 min to obtain the supernatant as the source of the crude tyrosinase extract. The protein content in the supernatant was determined using a Bradford assay with BSA as the protein standard. Tyrosinase activity was then determined as follows: 1 mL of the reaction mixture contained 50 mM of phosphate buffer (pH 6.8), 2.5 mM of l-DOPA, the tested drug, and 500 μg of the supernatant protein, and was incubated at 37 °C for 15 min, following which the dopachrome formation was monitored by measuring absorbance at a wavelength of 475 nm.

### 3.7. Statistical Analysis

All the data in the present study were obtained as averages of experiments that were performed in triplicate and are expressed as means ± S.D. Statistical analysis was performed by the Student’s *t* test. A value of *p* < 0.001 was considered to be statistically significant.

## 4. Conclusions

In conclusion, our results clearly demonstrated that searching new melanogenesis inhibitors from generic drugs is an alternative approach to develop new depigmenting agents in cosmeceuticals. From the results of the present study, cinnarizine and trazodone may be useful as candidates as skin-whitening agents for the treatment of skin hyperpigmentation.

## Figures and Tables

**Table 1:** The generic drugs screened in this study. In the table, some selected drugs, which are the same generic drug but produced by different company with a different brand or commercial name, are listed as the same drug name. The positively screened drugs are shown in a bold font.

The generic drugs screened in this study				
Acarbose	Colchicine	Fexofenadine	Meloxicam	Pertiazem
Acemetacin	Cortisone	Fluconazole	Mepenzolate	Phenytoin
Acetaminophen	Coumadin	Fludiazepam	Mephenoxalone	Pioglitazone
Aldioxa	Cyprohetadine	Flunarizine	Mequitazine	Piracetam
Allopurinol	Dacoton	Fluoxetine	Metformin	Polaramine
Alusa	**Danazol**	Formoterol	Methimazole	Ranitidine
Ambroxol	Dexamethasone	Furosemide	Methocarbamol	Rifater
Amlodipine	Dexchlorpheniramine	Gemfibrozil	Methotrexate	Ritampin
Amoxicillin	Diclofenac potassium	Ginkgoflavon	Metoclopramide	Rosiglitazone
Apresoline	Dicloxacillin	Glibenclamide	Metronidazole	Selegiline
**Aspirin**	Dihydroergotoxine	Glimepiride	Mexiletine	Sennoside
Atenolol	Dimethicone	Glipizide	**Miconazole**	Silymarin
Baclofen	Diovan	Glucosamine	Minocycline	Simvastatin
Benzbromarone	Diphenidol	**Homochlorcyclizine**	Mosapride	Songora
Benzonatate	Dipyridamole	Hydralazine	Nalidixic acid	Souriree
Bethanechol	Ditiazem	Hydroxychloroquine	Naproxen	Spironolaotone
Biorix	Domperidol	Hydroxyzine	Narcaricin	Strocain
Bisacodyl	Doxazosin	Hyoscyamine	Neomycin	Sulfinpyrazone
Bismuth	Doxycycline	Ibuprofen	Nicardipine	Sulindac
Bisoprolol	Doxymycin	Imdur	Nicergoline	Tamoxifen
Bromhexine	Elistin	Indapamide	Nicorandil	Terazosin
Captopril	Enalapril	Indomethanoin	Nifedipine	Theophyllin
Carvedilol	Entacapone	Iodopropylidene	Nifuroxazide	Thyroxine
Cefadroxil	Enzdase	Isoniazid	Nimodipine	**Trazodone**
Celecoxib	Eprazine	Isosorbide	Nitroxoline	Trihexyphenidy
Cephalexin	Ergonovine	Ketoconazole	Nystatin	Trimethoprim
Cetirizine	Erythromycin	Ketorolac	Ofloxacin	Tsurupinate
Cimetidine	Escitalopram	Levamisole	Oxatomide	Uliden
**Cinnarizine**	Ethambutol	Loperamide	Pantoprazole	Ultracet
Ciprofloxacin	Famotidine	Loratadine	Pecolin	Valproic acid
Clindamycin	Felodipine	Mapirocin	Pentoxifylline	Verapamil
Clonazepam	Fenoterol	Mebendazole	Peptidin	Warfarin
